# Sustainable Nanomedicine: Enhancement of Asplatin’s Cytotoxicity In Vitro and In Vivo Using Green-Synthesized Zinc Oxide Nanoparticles Formed via Microwave-Assisted and Gambogic Acid-Mediated Processes

**DOI:** 10.3390/molecules29225327

**Published:** 2024-11-12

**Authors:** Hatem A. F. M. Hassan, Nada K. Sedky, Mohamed S. Nafie, Noha Khalil Mahdy, Iten M. Fawzy, Toka Waleed Fayed, Eduard Preis, Udo Bakowsky, Sherif Ashraf Fahmy

**Affiliations:** 1 Medway School of Pharmacy, University of Kent, Chatham Maritime, Kent ME4 4TB, UK; h.a.hassan@kent.ac.uk; 2 Department of Pharmaceutics and Industrial Pharmacy, Faculty of Pharmacy, Cairo University, Cairo 11562, Egypt; nkmahdy@gmail.com; 3 Department of Biochemistry, School of Life and Medical Sciences, University of Hertfordshire Hosted by Global Academic Foundation, R5 New Garden City, New Administrative Capital, Cairo 11835, Egypt; nadasedky22@gmail.com; 4 Department of Chemistry, College of Sciences, University of Sharjah, Sharjah 27272, United Arab Emirates; mohamed.elsayed@sharjah.ac.ae; 5 Chemistry Department, Faculty of Science, Suez Canal University, Ismailia 41522, Egypt; 6 School of Life and Medical Sciences, University of Hertfordshire Hosted by Global Academic Foundation, R5 New Garden City, New Administrative Capital, Cairo 11835, Egypt; 7 Department of Pharmaceutical Chemistry, Faculty of Pharmacy, Future University in Egypt, Cairo 11835, Egypt; 8 Department of Pharmaceutics and Biopharmaceutics, University of Marburg, Robert-Koch-Str. 4, 35037 Marburg, Germany

**Keywords:** cancer therapy, asplatin, zinc oxide nanoparticles, drug delivery, green synthesis, triple-negative breast cancer

## Abstract

Chemoresistance encountered using conventional chemotherapy demands novel treatment approaches. Asplatin (Asp), a novel platinum (IV) prodrug designed to release cisplatin and aspirin in a reductive environment, has demonstrated high cytotoxicity at reduced drug resistance. Herein, we investigated the ability of green-synthesized nanocarriers to enhance Asp’s efficacy. Zinc oxide nanoparticles (ZnO-NPs) were synthesized using a green microwave-assisted method with the reducing and capping agent gambogic acid (GA). These nanoparticles were then loaded with Asp, yielding Asp@ZnO-NPs. Transmission electron microscopy was utilized to study the morphological features of ZnO-NPs. Cell viability studies conducted on MDA-MB-231 breast cancer cells demonstrated the ability of the Asp@ZnO-NPs treatment to significantly decrease Asp’s half-maximal inhibitory concentration (IC_50_) (5 ± 1 µg/mL). This was further demonstrated using flow cytometric analysis that revealed the capacity of Asp@ZnO-NPs treatment to significantly increase late apoptotic fractions. Furthermore, in vivo studies carried out using solid Ehrlich carcinoma-bearing mice showed significant tumor volume reduction with the Asp@ZnO-NPs treatment (156.3 ± 7.6 mm^3^), compared to Asp alone (202.3 ± 8.4 mm^3^) and untreated controls (342.6 ± 10.3 mm^3^). The histopathological analysis further demonstrated the increased necrosis in Asp@ZnO-NPs-treated group. This study revealed that Asp@ZnO-NPs, synthesized using an eco-friendly approach, significantly enhanced Asp’s anticancer activity, offering a sustainable solution for potent anticancer formulations.

## 1. Introduction

Induced resistance against conventional chemotherapy and the ensuing high recurrence rates necessitate the exploration of novel cancer treatment approaches [1,2,3]. Platinum-based anticancer drugs are among the most extensively used chemotherapies [4]. Platinum-contained chemotherapies exhibit their anticancer activity through the formation of DNA cross-links that inhibit the transcription process and induce apoptotic events. Nevertheless, the observed side effects associated with platinum-based therapies, such as cisplatin and oxaliplatin, highlight the need for novel, efficient derivatives [5].

Asp, a platinum (IV) aspirin-conjugated prodrug that releases cisplatin under reductive conditions, has exhibited markedly higher cytotoxicity against tumor cells and reduced drug resistance in cisplatin-resistant cells [6]. Accordingly, the utilization of Asp could offer enhanced cytotoxic effects at reduced doses. Furthermore, growing evidence suggests that low-dose aspirin has anticancer activity, particularly in reducing long-term risk, cancer cell proliferation, and metastatic potential [7,8,9,10]. For instance, Gu et al. previously reported the aspirin’s ability to induce anti-proliferative and apoptotic effects in PIK3CA-mutant colon cancer cells [11]. To this end, the Asp-mediated intracellular aspirin and cisplatin release could provide synergistically derived cytotoxicity. The cytotoxic effects of Asp against cisplatin were previously evaluated in comparative studies. A lower IC_50_ was previously exhibited by Asp than cisplatin against various cancer cell types, including MCF-7 breast cancer cells and A549 lung cancer cells [6]. Similarly, Asp previously showed a higher ability to reduce the HeLa cells viability compared to cisplatin or oxoplatin [12]. Additionally, Asp treatment showed higher antitumor activity and lower toxicity than cisplatin in HepG2 tumor-bearing mice [13]. However, the safe application of Asp in clinical settings demands achieving effective therapeutic outcomes at low administration doses. Hence, incorporating Asp into a nanoparticle-based delivery system could address these limitations by intensifying the cytotoxicity against cancer cells at reduced doses.

Nanosized carriers have demonstrated the capacity to enhance the therapeutic outcome of the incorporated therapeutic agents against various pathological conditions [14,15,16,17,18]. Additionally, nanoformulations have been utilized in oncology to offer efficient delivery that could enhance the efficacy of anticancer agents and reduce their side effects [19,20,21,22,23,24]. Zinc oxide nanoparticles (ZnO-NPs) have exhibited the ability to improve the cellular uptake of the loaded therapeutic cargo and reduce the resistance induced against chemotherapies [25,26]. Interestingly, Sharma et al. previously reported the increased uptake of doxorubicin-loaded onto ZnO-NPs following the incubation with MCF-7 cells [27]. Liu et al. also reported the ability of the ZnO-NPs to increase the cell uptake of the incorporated doxorubicin and reduce its efflux following the internalization by MCF-7R cells [28]. In light of these findings, the features ZnO-NPs possess as a nanocarrier could improve the deliverability of incorporated Asp and potentiate its cytotoxic activities.

Several methods have been reported for the synthesis of ZnO-NPs. For instance, using the hydrothermal method, ZnO-NPs could be obtained through the crystallization from a ZnO solution under pressurized and high-temperature conditions [29]. ZnO-NPs can also be produced using the chemical vapor deposition method through the reaction between zinc vapor and oxygen under controlled conditions [30]. Additionally, the precipitation method is employed to prepare ZnO-NPs by reacting zinc salts with a base in an aqueous solution under controlled conditions, forming precipitates that, upon subsequent calcination, yield ZnO-NPs [31]. The green-synthesized nanoparticles (NPs) have attracted increasing interest as a sustainable approach that utilizes natural products as well as plant-derived reducing and capping agents. Gambogic acid (GA) is a natural prenylated xanthone compound derived from the resin of *Garcinia hanburyi* tree [32]. Previous studies have illustrated the potential of *Garcinia extracts* to be utilized in the green synthesis of inorganic nanoparticles [33,34,35]. Using this eco-friendly method, GA could facilitate the synthesis of ZnO-NPs through the reduction of metal ions and the formation of coordination complexes, leading to the nucleation and growth of the NPs. GA may also act as a capping agent to stabilize the NPs and control their size and shape. The green synthesis approach offers environmentally friendly conditions that can be conducted at room temperature without utilizing hazardous chemicals and high-energy processes [36,37,38]. Hence, exploiting GA in the biosynthesis of ZnO-NPs could offer an eco-friendly cancer therapeutic approach. In addition, previous studies have reported the ability of microwave-assisted radiation to nanosize the particles, yielding uniform and monodispersed nanoparticles [39]. Thus, the combination of microwave-assisted synthesis with green chemistry principles could constitute an attractive strategy for the efficient development of biocompatible and therapeutically effective nanoparticles.

In this study, we hypothesized that the incorporation of Asp into ZnO-NPs synthesized using GA via microwave-assisted methods could result in a formulation with superior anticancer activity compared to free Asp. GA was utilized in the green synthesis of ZnO-NPs under microwave-assisted radiation conditions. The obtained ZnO-NPs were incorporated with Asp drug, yielding Asp@ZnO-NPs, and then characterized. Afterwards, the capability of the obtained ZnO-NPs to potentiate the Asp anticancer activities was evaluated against MDA-MB-231 breast cancer cells in vitro and in solid Ehrlich carcinoma-bearing mice. Through the development of green-synthesized chemotherapies-loaded nanoparticles, the outcomes of this study could provide scalable and environmentally sustainable solutions for producing potent anticancer formulations.

## 2. Results and Discussion

### 2.1. Physicochemical Characterization of the Prepared Nanoparticles

**Figure 1:**
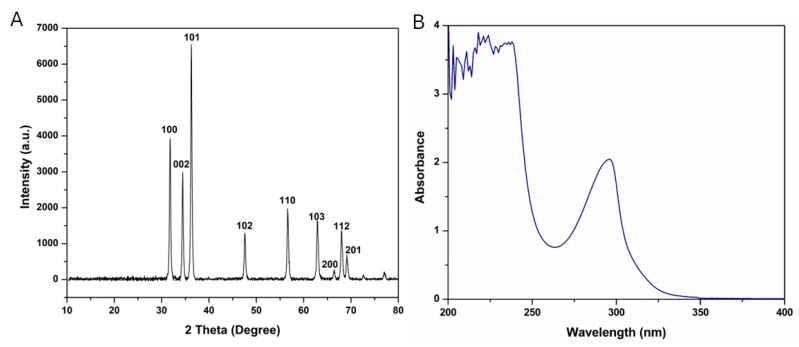
(**A**) XRD pattern of ZnO-NPs and (**B**) UV-Vis absorbance spectrum of Asp@ZnO-NPs.

ZnO-NPs were biosynthesized involving gambogic acid (GA) as a natural reductant combined with microwave energy as a sustainable and eco-friendly method. The XRD spectrum of the green-synthesized ZnO-NPs (Figure 1A) showed nine diffraction peaks at 31.79°, 34.45°, 36.28°, 47.59°, 56.65°, 62.92°, 66.45°, 68.03°, and 69.17°, which were recognized as (1 0 0), (0 0 2), (1 0 1), (1 0 2), (1 1 0), (1 0 3), (2 0 0), (1 1 2), and (2 0 1) reflections, respectively. The obtained sharp and strong XRD peaks are typical for ZnO fine crystalline structure, according to the Joint Committee on Powder Diffraction Standards card number 79-2205 [40,41]. The average crystallite size of the ZnO-NPs was computed from the full width at half maximum (FWHM) of the diffraction peaks obtained using Debye–Sherer’s equation, i.e., Equation (1), and was found to be 24.50 nm. This nanoscale size demonstrated the ability of the NPs to passively enter the leaky tumor cells, exerting their therapeutic effects [20]. The 2θ, FWHM, and nanoparticles’ diameter from the XRD pattern of the ZnO-NPs are presented in Table 1.

Asp was conjugated onto the ZnO-NPs, resulting in Asp@ZnO-NPs with EE% of 79.5% ± 5.7%, suggesting efficient Asp incorporation. The surface charge of nanoparticles is a critical factor influencing their electrostatic repulsion. Repulsive forces that exceed the attractive Van der Waals forces could reduce the tendency for nanoparticle aggregation, thereby promoting dispersion stability [42]. To evaluate the surface charge of the synthesized ZnO-NPs and Asp@ZnO-NPs, Zeta Potential (ZP) analysis was conducted. The analysis indicated high negative ZP values, suggesting that the nanoparticle colloids exhibit high stability upon dispersion. The ZnO-NPs exhibited a ZP of −26.7 ± 2.4 mV, which is attributed to the presence of capping molecules, such as GA, on the nanoparticle surfaces. This observation aligns with previous findings [43]. On the other hand, the Asp@ZnO-NPs displayed a lower ZP of −15.8 ± 1.9 mV upon loading with Asp, which can be attributed to the cationic nature of Asp [44,45,46]. In addition, the UV-Vis absorbance spectrum of Asp@ZnO-NPs showed a peak at 295 nm (Figure 1B) that was blue-shifted compared to ZnO-NPs that showed an absorbance spectrum at 369 nm and was red-shifted as compared to free Asp that showed an absorbance spectrum at 282, as presented in Figure S3 in the Supplementary Materials. Our findings correspond well with previous reports [45,47]. The observed blue shift could be interpreted as an indication of successful Asp loading onto the ZnO-NPs [48].

**Figure 2:**
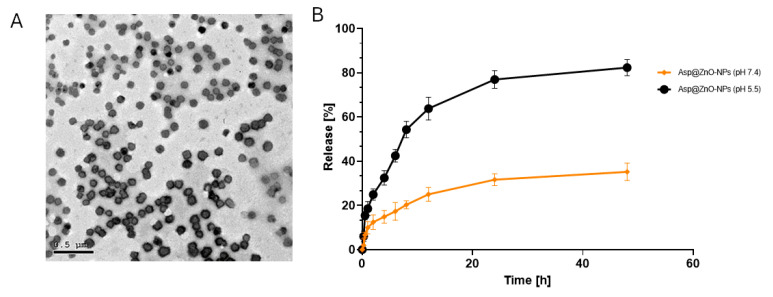
(**A**) TEM micrograph of Asp@ZnO-NPs and (**B**) release profile of Asp from Asp@ZnO-NPs at pH 7.4 and pH 5.2 over 48 h. Results represent the mean ± S.D.

Transmission electron microscopy (TEM) was utilized to visualize the morphology of the synthesized Asp@ZnO-NPs, as illustrated in Figure 2A. The prepared NPs unveiled a spherical morphology with flat surfaces and trivial agglomerations. The TEM examination of Asp@ZnO-NPs shows a size range of 55–85 nm. The increase in size of Asp@ZnO-NPs, as compared to the size of plain ZnO-NPs (obtained from the XRD study), was an extra tool that confirmed the successful loading of Asp onto ZnO-NPs.

### 2.2. The Release Profile In Vitro

The release profile of Asp from Asp@ZnO-NPs was evaluated using the dialysis sac method under physiological conditions (pH 7.4) and acidic conditions mimicking tumor tissue (pH 5.2) (Figure 2B) [43]. At pH 7.4, the release of Asp from Asp@ZnO-NPs showed a slow and sustained release profile. The initial release was minimal, with less than 20% of Asp released within the first 10 h. The release gradually increased over the next 48 h, reaching approximately 25%. The observed slow and sustained release could decrease the systemic toxicity, maintain therapeutic drug levels, and reduce the dosing frequency.

At pH 5.2, Asp release from Asp@ZnO-NPs exhibited a rapid release profile compared to that assessed at pH 7.4. Within the first 10 h, the release at pH 5.2 reached approximately 50%. The release continued to increase rapidly, reaching around 80% by the end of the 48 h period. This significantly higher release rate in acidic conditions suggests that Asp@ZnO-NPs could be more effective in releasing the drug in acidic tumor microenvironments [49]. The rapid release in these conditions can enhance the cytotoxic effects on tumor cells, while the sustained release in physiological conditions may reduce systemic exposure and the associated adverse effects on normal tissues [50].

### 2.3. In Vitro Assessment

**Figure 3:**
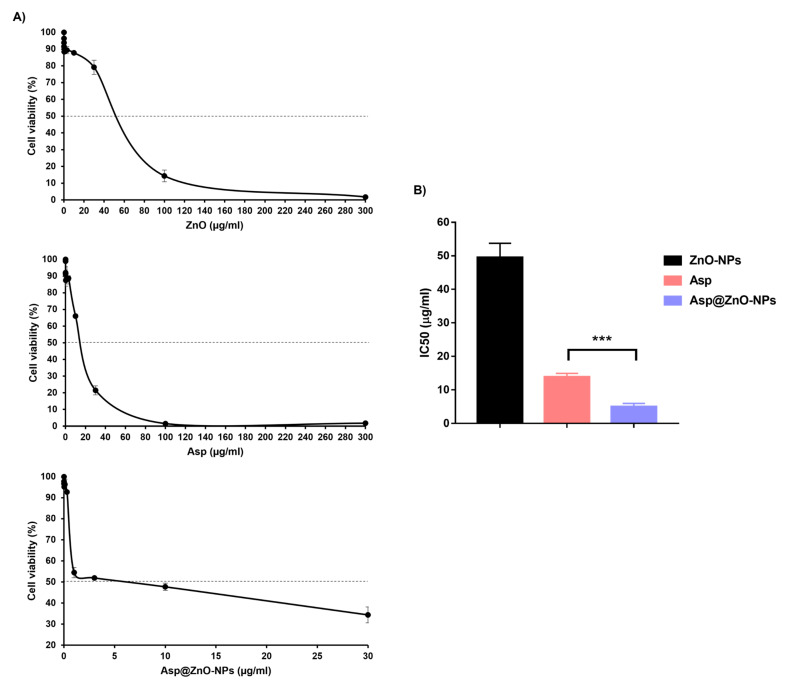
Assessment of the MDA-MB-231 cells’ viability in vitro. (**A**) Dose–response curves showing the viability of the MDA-MB-231 cells treated with ZnO-NPs, Asp, or Asp@ZnO-NPs at different concentrations for 48 h. (**B**) The IC_50_ of ZnO-NPs, Asp, or Asp@ZnO-NPs following the incubation with the MDA-MB-231 cells. Results represent the mean value ± S.D. (n = 3). *** *p* < 0.001.

The main aim of this study was to assess the Asp’s effectiveness following the incorporation onto ZnO-NPs, with an average Asp content of 36.8 wt% in the Asp@ZnO-NPs. The effect of ZnO-NPs, Asp, and Asp@ZnO-NPs on MDA-MB-231 breast cancer cells’ viability after 48 h of incubation was evaluated using the SRB assay. The Asp treatment showed an IC_50_ value of 13.81 ± 1.133 µg/mL (Figure 3). Interestingly, the IC_50_ detected following treatment with Asp@ZnO-NPs was 5 ± 1 µg/mL. The significantly lower IC_50_ of Asp@ZnO-NPs compared to Asp alone (*p* < 0.05) suggested that the cytotoxic effects of Asp were boosted when delivered using ZnO-NPs.

**Figure 4:**
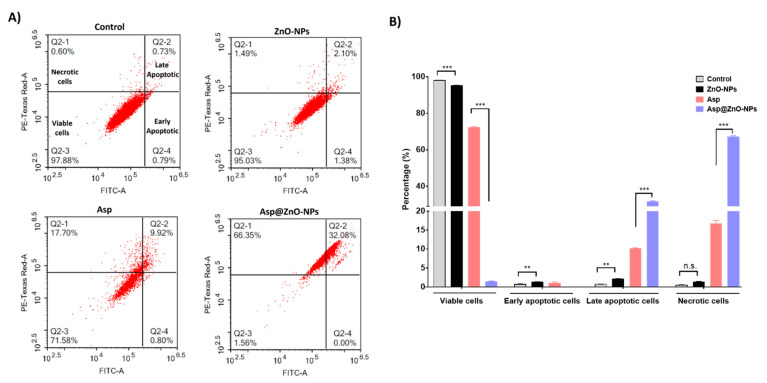
Assessment of MDA-MB-231 cell apoptosis using Annexin V-FITC apoptosis detection kit. (**A**) Flow cytometric analysis of MDA-MB-231 cells incubated with ZnO-NPs, Asp, or Asp@ZnO-NPs for 48 h. Untreated cells were included as a control. (**B**) Percentage of the necrotic (Q2-1), late apoptotic (Q2-2), viable (Q2-3), and early apoptotic (Q2-4) fractions of the MDA-MB-231 cells treated with ZnO-NPs, Asp, or Asp@ZnO-NPs. Results represent the mean value ± S.D. (n = 3). ** *p* < 0.01; *** *p* < 0.001. n.s. is non-significant.

Furthermore, the apoptotic and necrotic fractions of MDA-MB-231 cells exposed to ZnO-NPs, Asp, or Asp@ZnO-NPs for 48 h were assessed (Figure 4). Treatment with Asp alone significantly reduced cell viability and increased the late apoptotic and necrotic fractions. However, the incorporation of Asp onto ZnO-NPs significantly amplified the Asp-induced apoptosis and necrosis, leading to a substantial reduction in viable cell fraction as well as a marked increase in late apoptotic and necrotic cell fractions compared to Asp alone or untreated cells (*p* < 0.001).

The observed enhancements in the Asp anticancer activity could be attributed to several factors. ZnO-NPs may facilitate better cellular uptake of Asp, consequently increasing the intracellular concentration of the drug [27,28]. Moreover, it was previously reported that ZnO-NPs could limit the ATP-binding cassette transporters’ activity, which is responsible for the efflux of drugs from cancer cells [51]. By potentially inhibiting these transporters, ZnO-NPs could help retain more drug molecules within the cells. Furthermore, the sustained release of Asp from the ZnO-NPs could maintain therapeutic drug levels within the cancer cells over a prolonged period, thus enhancing its efficacy [27,28]. The significant enhancement in the Asp’s cytotoxic effects mediated via the ZnO-NPs observed in our study suggests that Asp@ZnO-NPs could potentially be an effective anticancer formulation.

### 2.4. In Vivo Assessments

#### 2.4.1. Antitumor Activity

**Figure 5:**
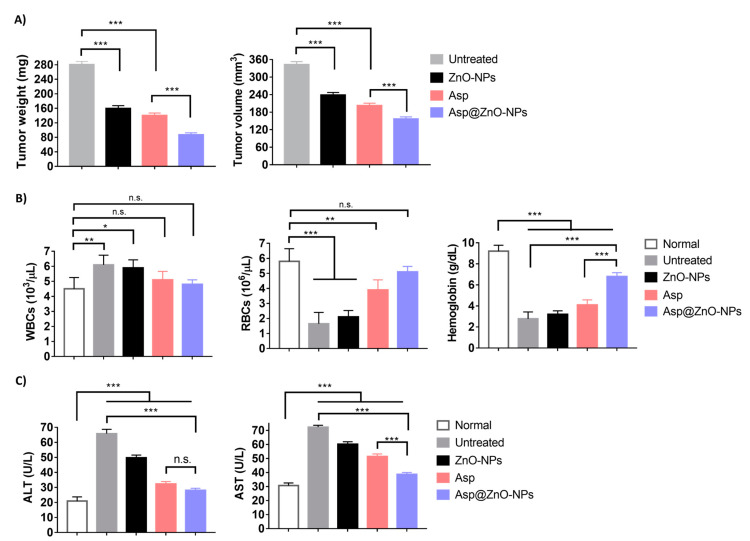
Assessment of antitumor effects induced in solid Ehrlich carcinoma-bearing mice. Female Swiss albino mice subcutaneously inoculated with solid Ehrlich carcinoma were divided into four groups (n = 5). The tumor-bearing mice were left untreated or intraperitoneally injected with ZnO-NPs, Asp, or Asp@ZnO-NPs at 6 mg/kg body weight every other day. Normal Swiss albino mice (n = 5) were included as a control. (**A**) The weight and volume of the tumors excised from the tumor-bearing mice on day 24 post-tumor inoculation. (**B**) Hematological parameter assessment, showing the WBC, RBC, and hemoglobin levels in blood samples collected from normal or tumor-bearing mice on day 24. (**C**) Serum levels of the liver enzymes ALT and AST in blood samples collected from the mice on day 24. Results represent the mean value ± S.D. (n = 5). * *p* < 0.05; ** *p* < 0.01; *** *p* < 0.001. n.s. is non-significant.

The antitumor efficacy was studied in female Swiss albino mice with solid Ehrlich carcinoma implanted subcutaneously. The findings showed that tumor-bearing mice left untreated had the highest average tumor size (342.6 ± 10.3 mm^3^), verifying the aggressive behavior of solid Ehrlich carcinoma in the absence of treatment [52] (Figure 5A). The use of Asp led to a notable decrease in tumor size (202.3 ± 8.4 mm^3^), whereas treatment with Asp@ZnO-NPs showed the most significant reduction in tumor volume (156.3 ± 7.6 mm^3^). A comparable pattern was noted across the different treatments when the weight of the removed tumor was assessed on day 24 after tumor inoculation. In addition, histopathological examination corroborated these findings, showing extensive necrosis and reduced cellular and nuclear pleomorphism in the mice group treated with Asp@ZnO-NPs (Figure 5A). The untreated group exhibited dense clusters of tumor cells with high nuclear pleomorphism, indicating aggressive tumor growth. However, the Asp@ZnO-NPs-treated group showed a marked reduction in cellular density and nuclear pleomorphism, with increased areas of necrosis [53]. The histopathological findings supported the quantitative data, demonstrating that the Asp@ZnO-NP treatment leads to significant tumor regression. The Asp’s ability to significantly retard tumor growth highlights the dual anticancer activity of its cisplatin and aspirin content [12,54,55,56]. However, consistent with the in vitro results, the combination of Asp with ZnO-NPs augmented the antitumor effects, which could be due to the synergistic effects. The ZnO-NPs likely enhanced the therapeutic efficacy of Asp by improving cellular uptake and intracellular retention, leading to more effective tumor cell eradication [25,27,28,51]. Additionally, the extended release of Asp from the Asp@ZnO-NPs observed in the in vitro studies further supports this enhanced efficacy [27,28]. The sustained release of Asp from ZnO-NPs could allow for prolonged exposure of the tumor cells to the therapeutic agent, maximizing its antitumor activity.

#### 2.4.2. Assessment of Hematological and Biochemical Parameters

The hematological parameters of the female Swiss albino mice were assessed on day 24 post-tumor inoculation (Figure 5B). The average Hb level in normal mice was 9.2 ± 0.57 g/dL. Mice with tumors that were not treated experienced a significant drop in Hb levels, measuring at 2.78 ± 0.65 g/dL. Asp-treated mice demonstrated slightly higher Hb levels, reaching 4.1 ± 0.48 g/dL. The Asp@ZnO-NPs treatment markedly increased Hb levels to 6.8 ± 0.36, approaching normal levels. The reduction in Hb levels in untreated tumor-bearing mice indicated significant anemia, a common symptom associated with advanced cancer due to the reduced ability to produce enough RBCs to counteract the tumor burden [57]. Asp treatment showed a notable improvement in Hb levels, indicating its efficacy in alleviating the hematological impact of the tumor [58].

Utilizing Asp@ZnO-NPs for treatment showed significant therapeutic benefits, nearly restoring Hb levels to normal. Additionally, the tumor-carrying mice that did not receive treatment exhibited a significant decrease in RBCs of 1.65 ± 0.75 × 10^6^/μL, in contrast to the healthy mice with a red blood cell count of 5.8 ± 0.84 × 10^6^/μL. The Asp significantly raised the RBC to 3.9 ± 0.67 × 10^6^/μL. However, the Asp@ZnO-NPs treatment restored RBC count to near-normal levels at 5.1 ± 0.36 × 10^6^/μL. The significantly reduced RBC count in untreated tumor-bearing mice confirmed the presence of severe anemia. The Asp@ZnO-NPs showed the highest improvement, restoring the RBC count to near-normal levels.

Mice treated with Asp@ZnO-NPs exhibited a WBC count of 4.8 ± 0.3 × 10^6^/μL, comparable to levels detected in healthy mice. The near-normal WBC count in the Asp@ZnO-NPs-treated group indicated a substantial reduction in tumor-associated inflammation. This observation aligned with the well-established anti-inflammatory effects of aspirin, one of the Asp’s components [59]. On the contrary, untreated tumor-bearing mice showed a marked increase in WBC count that could be due to the inflammatory reaction induced by the established tumor [60].

The significant improvements in Hb levels, RBC counts, and normalization of WBC counts demonstrate the comprehensive benefits of the Asp@ZnO-NPs treatment, highlighting its potential as a potent anticancer therapy.

Moreover, untreated solid Ehrlich carcinoma-inoculated mice showed significantly elevated levels of ALT and AST, indicating substantial liver damage due to tumor growth [61,62] (Figure 5C). Nevertheless, the mice group treated with Asp@ZnO-NPs showed ALT and AST levels that were significantly lower than those detected in untreated tumor-bearing mice, suggesting reduced liver damage.

**Figure 6:**
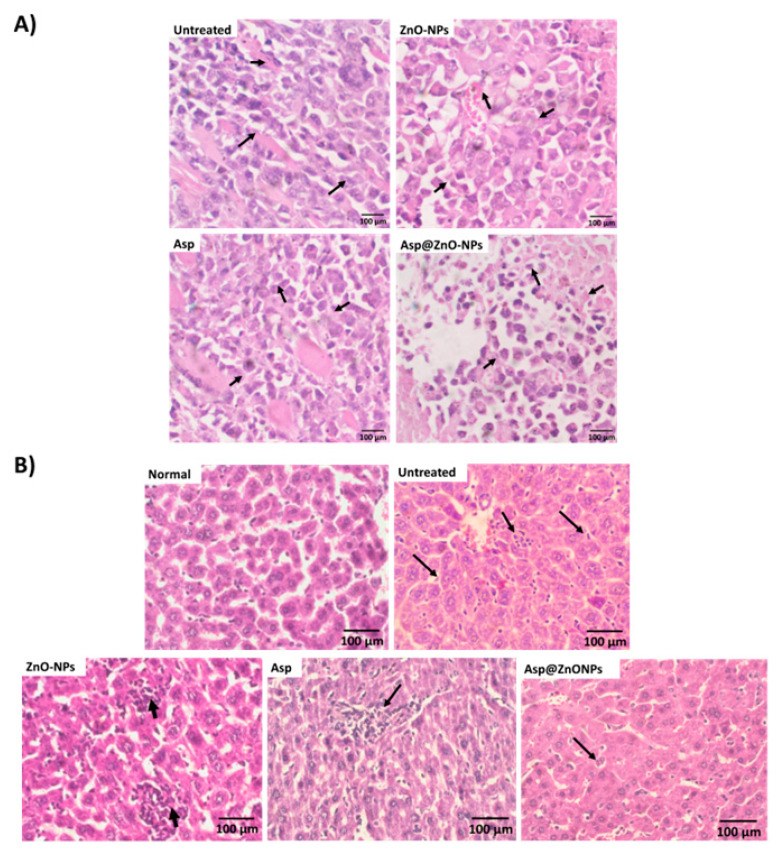
Histopathological examinations of (**A**) tumor tissues and (**B**) liver tissues excised from Swiss albino mice that are stained with hematoxylin and eosin (400×).

The untreated tumor tissue showed densely packed cells with intact cellular architecture (Figure 6A). Tumor tissue collected from mice injected with ZnO-NPs exhibited signs of structural disorganization; however, the extent of tissue damage was limited, and the overall structure remained largely intact. In contrast, the tumor samples excised from mice treated with Asp@ZnO-NPs displayed the most extensive signs of cellular destruction, with areas of severe apoptosis characterized by condensed nuclei and cell shrinkage. Compared to the Asp alone treatment, the Asp@ZnO-NPs treatment significantly amplified the cytotoxic effect, leading to more extensive necrosis throughout the tumor tissue.

The histopathological analysis of liver tissues further demonstrated the hepatoprotective effects of Asp@ZnO-NPs in the mice bearing solid Ehrlich carcinoma (Figure 6B). The untreated group exhibited severe liver damage characterized by extensive necrosis and inflammation. This damage is likely due to the tumor burden and associated systemic effects. Asp treatment improved the liver histology, suggesting its potential to reduce tumor-induced liver damage. Treatment with Asp@ZnO-NPs resulted in a hepatoprotective effect, with minimal necrosis, inflammation, and well-preserved liver architecture [63]. These findings were consistent with the observed biochemical markers of liver function, where the Asp@ZnO-NPs-treated group had the lowest levels of liver enzymes ALT and AST. The obtained results suggest that the Asp@ZnO-NPs formulation not only enhanced the therapeutic efficacy against tumors but also provided significant protection to liver tissues, reducing the systemic toxicity often associated with cancer treatments.

## 3. Materials and Methods

### 3.1. Materials

Dulbecco’s Modified Eagle’s Medium (DMEM), fetal bovine serum, penicillin, streptomycin, tris (hydroxymethyl)aminomethane, and Sulforhodamine B colorimetric (SRB) assay kit were obtained from Lonza (Basel, Switzerland). Annexin V-FITC apoptosis detection kit was purchased from Abcam Inc. (Cambridge, UK). Aspartate transaminase (AST) and alanine transaminase (ALT) assay kits were obtained from Instrumentation Laboratory SpA (Milan, Italy) and Inova Diagnostics (San Diego, CA, USA). All of the other chemicals were sourced from Sigma Aldrich (St. Louis, MO, USA).

### 3.2. Microwave-Assisted Green Synthesis of ZnO-NPs

Following a previously reported procedure, the ZnO-NPs were prepared with some modifications [39]. In 20 mL of deionized water, GA and zinc acetate dihydrate at a 1:10 ratio were mixed, and the yielded mixture was then exposed to 10 cycles of microwave irradiation at 30 s per cycle for a total duration of 5 min. Following microwave exposure, a metal hydroxide paste was produced. Finally, the obtained paste was subsequently calcined in a muffle furnace at 600 °C for 5 h, yielding ZnO-NPs.

### 3.3. Preparation of Asp@ZnO-NPs

ZnO-NPs were suspended in 15 mL of deionized water, and the mixture was stirred for 15 min. Asp was synthesized as described in the Supplementary Materials and presented in Figures S1 and S2, following our previously reported method [6]. An aqueous solution of Asp was then added dropwise under continuous stirring. The Asp and ZnO-NPs mixture was bath-sonicated for 15 min, centrifuged at 12,000 rpm for 1 h, and subsequently dialyzed against deionized water for 24 h. The colloidal mixture was freeze-dried to obtain dry Asp@ZnO-NPs.

### 3.4. Physicochemical Characterization of the Prepared Nanoparticles

#### 3.4.1. Zeta Potential (ZP) Analysis

To evaluate the charge of the synthesized ZnO-NPs and Asp@ZnO-NPs, Zeta Potential (ZP) measurements were conducted using a Zetasizer Nano ZS (Malvern Instruments, Herrenberg, Germany) [40,41]. Initially, the nanoparticles were dispersed in distilled water and sonicated for 5 min to prevent aggregation and ensure proper dispersion. The measurement parameters were configured as follows: refractive index: 1.33; water viscosity: 0.887 mPa·s; HeNe laser power: 10 mW; wavelength: 633 nm; and backscatter detector angle: 173°. Zeta Potential measurements were performed using a laser Doppler velocimeter (Malvern Instruments, Herrenberg, Germany). All experiments were conducted in triplicates, and standard deviations (SD) were calculated.

#### 3.4.2. Ultraviolet–Visible Spectroscopy (UV-Vis)

UV-Vis spectrophotometry (Peak instruments T-9200, Houston, TX, USA) was employed to acquire the absorption spectrum of the synthesized ZnO-NPs within the UV-Vis range. Prior to measurement, the dry samples were suspended in distilled water and dispersed by sonication. The UV-Vis absorption spectra were then recorded over a wavelength range of 200 to 700 nm.

### 3.5. X-Ray Diffraction Analysis (XRD)

The Bruker D8 Discover X-ray diffractometer (Karlsruhe, Germany) was employed to detect the X-ray diffraction patterns of ZnO-NPs. The applied parameters were set as follows: electric potential: 40 KV; electric current: 40 mA; CuKα radiation wavelength (λ): 1.5406 Å; 2 Theta (θ) scale: 10–80°; and step size for phase recognition: 0.02.

Debye–Scherer’s equation, i.e., Equation (1), was used to calculate the average diameter of the ZnO-NPs [16,19].

(1) $$D=\frac{0.9\lambda }{\beta \cos\theta }$$

The symbols in Equation (1) refer to the following: D: ZnO-NPs’ diameter; λ: CuKα radiation wavelength; β: full width at half maximum (FWHM) of the respective diffraction peak; and θ: Bragg diffraction angle.

### 3.6. High-Resolution Transmission Electron Microscopy (HRTEM)

High-Resolution Transmission Electron Microscopy (JEOL JEM-2100, Musashino, Akishima, Tokyo, Japan, functioning at 140 kV) was employed to visualize the morphology of the NPs. The synthesized Asp@ZnO-NPs (diluted with deionized water in a ratio of 1:2) were stained with 2% aqueous phosphotungstic acid and eventually dried over a carbon-coated copper 200-mesh grid for imaging.

### 3.7. Entrapment Efficiency (EE%)

The EE% of Asp in Asp@ZnO-NPs was determined by centrifuging 2 mL of the developed NPs at 14,000 rpm and 4 °C for 2 h using an ultracentrifuge (Hermle Z 326 K, Labortechnik GmbH, Wehingen, Germany). Subsequently, the centrifugate was ultrafiltrated to remove free Asp, which was quantified using HPLC as described in our previous study [6]. Equation (2) was employed to calculate the EE% [64]:

(2) $$\mathrm{EE}\%=\frac{\mathrm{Total}\;\mathrm{amount}\;\mathrm{of}\;\mathrm{Asp}-\mathrm{Free}\;\mathrm{Asp}}{\mathrm{Total}\;\mathrm{amount}\;\mathrm{of}\;\mathrm{Asp}}\times 100$$

### 3.8. In Vitro Release

The dialysis sac method was used to assess the release percentage of Asp from Asp@ZnO-NPs at pH 7.4 (mimicking physiological conditions) and pH 5.2 (mimicking tumor tissue). Briefly, a known volume of NPs (0.5 mL) was inserted into a dialysis sac (14 kDa cut-off), submerged into a vessel containing 10 mL of either acetate buffer (pH 5.5) or PBS (pH 7.4), and shaken at 37 ± 0.5 °C for 48 h. The released Asp was quantified using HPLC, as described in our previous study [6], where 1 mL of the release medium was collected at precise time intervals and immediately replaced with 1 mL of warmed buffer. The release percentage of Asp was estimated using Equation (3):

(3) $$\mathrm{Release}\%=\frac{\mathrm{Amount}\;\mathrm{of}\;\mathrm{released}\;\mathrm{Asp}}{\mathrm{Initial}\;\mathrm{amount}\;\mathrm{of}\;\mathrm{loaded}\;\mathrm{Asp}}\times 100$$

### 3.9. In Vitro Studies

#### 3.9.1. Cells and Culture Media

MDA-MB-231 breast cancer cells sourced from Nawah Scientific Inc., Cairo, Egypt, were cultured at 37 °C in a humidified atmosphere with 5% (*v*/*v*) CO_2_ in DMEM complete medium containing 100 mg/mL streptomycin, 100 units/mL penicillin, and 10% heat-inactivated fetal bovine serum.

#### 3.9.2. Cell Viability Studies

MDA-MB-231 cells were plated at a seeding density of 5000 cells in each well of 96-well plates and were cultured with ZnO-NPs, Asp, or Asp@ZnO-NPs for 48 h. Concentrations used ranged from 0.01 to 300 µg/mL for both ZnO-NPs and Asp and from 0.001 to 30 µg/mL for Asp@ZnO-NPs. The cells were fixed through incubation at 4 °C for 1 h in 150 μL of 10% complete media. After the complete media was removed, cells were washed five times with distilled water. Cell viability was determined using SRB assay, as outlined before [6]. The cells were exposed to SRB solution in the dark for a duration of 10 min. Following that, the cells were exposed to three rounds of 1% acetic acid rinses, left to dry in the air overnight, and subsequently dissolved in 150 μL of 10 mM Tris (hydroxymethyl)aminomethane buffer for each well. The BMGLABTECH^®^-FLUOstar Omega microplate reader from Ortenberg (Germany) was used to measure the absorbance at 540 nm.

#### 3.9.3. Determination of Cell Apoptosis

The apoptotic and necrotic fractions of treated MDA-MB-231 cells were assessed using an Annexin V-FITC apoptosis detection kit (Abcam Inc., Cambridge Science Park, Cambridge, UK). Briefly, MDA-MB-231 cells were left untreated or incubated with ZnO-NPs, Asp, or Asp@ZnO-NPs for 48 h. Afterwards, the incubated cells (1 × 10^5^) were harvested and rinsed twice with cold PBS (pH 7.4). Subsequently, the cells were incubated for 30 min with 0.5 mL of Annexin V-FITC/PI solution in the dark at room temperature. The cells were then processed using an ACEA Novocyte™ flow cytometer (ACEA Biosciences Inc., San Diego, CA, USA) to detect the FITC and PI fluorescence in the 12,000 events acquired for each sample. The ACEA NovoExpress™ software 1.6.2 (ACEA Biosciences Inc., San Diego, CA, USA) was then used to quantify the FITC- and/or PI-positive cells through quadrant analysis.

### 3.10. In Vivo Studies

#### 3.10.1. Animals

All procedures concerning the care and maintenance of the animals were conducted in accordance with international guidelines for animal research and were approved by the Bioethics and Animal Ethics Committee of the Faculty of Science, Suez Canal University (Approval number REC225/2023). Adult female Swiss albino mice obtained from the Faculty of Pharmacy, Suez Canal University, Ismailia, Egypt, with an average body weight of 18–23 g, were used in the study. The mice were maintained under a constant 12 h light/dark cycle in a controlled environment with a temperature of 22 ± 2 °C and humidity regulation. They had unrestricted access to standard laboratory mouse food and water.

#### 3.10.2. Assessment of Antitumor Activity

Solid Ehrlich carcinoma cells were sourced from the National Cancer Institute (Cairo University, Egypt). The tumor cell line was propagated in mice through serial intraperitoneal injections of 0.2 mL physiological saline containing 1 × 10^6^ viable cells that were administered over a 24 h period. The solid Ehrlich carcinoma cells were harvested 7 days after intraperitoneal implantation and diluted with saline to achieve a concentration of 5 × 10^6^ viable solid Ehrlich carcinoma cells/mL. A volume of 0.2 mL containing 1 × 10^6^ solid Ehrlich carcinoma cells was then intraperitoneally implanted into each normal mouse. Additionally, 1 × 10^6^ solid Ehrlich carcinoma cells per mouse were implanted subcutaneously into the right thigh of the hind limb. A group of mice (n = 5) that were not injected with solid Ehrlich carcinoma cells was included in the study as the normal control group. The mice inoculated with solid Ehrlich carcinoma cells were randomly assigned to four groups (n = 5). The first group served as the untreated control. The second group was intraperitoneally injected with ZnO-NPs at a dosage of 6 mg/kg body weight. The third group was treated with Asp at the same dosage and route. The fourth group was intraperitoneally administered with Asp@ZnO-NPs at 6 mg/kg body weight. At the end of the experiment, animals were anesthetized and sacrificed to evaluate antitumor activity and histopathological analysis.

#### 3.10.3. Hematological and Biochemical Analyses of Blood Samples

Blood samples were collected from the normal or the solid Ehrlich carcinoma-bearing mice (n = 5). The complete blood count (CBC), assessing hemoglobin concentration, red blood cell count, and white blood cell count, was carried out with the Abbott CELL-DYN^®^ 1800 automated hematology analyzer (USA) and a commercially available kit (Abbott Laboratories, Chicago, IL, USA). The collected serum samples were analyzed using commercial assay kits to quantify the levels of AST and ALT.

### 3.11. Histopathological Analysis

Liver and tumor tissue specimens from sacrificed mice were fixed in 10% formalin. The fixed samples were then dehydrated through an ascending series of ethyl alcohol and embedded in paraffin. Sections with a thickness of 5 µm were stained with hematoxylin and eosin and examined under light microscopy.

### 3.12. Statistical Analysis

Analysis using GraphPad Prism version 7.00 (Boston, MA, USA) was conducted. Results are reported as the average ± standard deviation (S.D.). Statistical differences were detected using one-way ANOVA and Bonferroni post-test.

## 4. Conclusions

The findings presented in this study suggest that the significant enhancement in Asp’s anticancer activities could be achieved upon the utilization of green-synthesized ZnO-NPs as a nanocarrier. The in vitro studies on MDA-MB-231 breast cancer cells revealed the ZnO-NPs’ capability to significantly lower Asp’s IC_50_ and increase its apoptotic effects. The in vivo studies in solid Ehrlich carcinoma-bearing mice corroborated these findings, showing significant tumor volume reduction and increased necrosis in the mice group administered with Asp@ZnO-NPs. Collectively, these findings highlight the potential of Asp@ZnO-NPs as a potent and sustainable anticancer formulation, offering a promising alternative to conventional chemotherapy.

## Figures and Tables

**Table 1:** 2θ, FWHM, and nanoparticles’ diameter obtained from the XRD pattern of the ZnO-NPs.

Peak #	2 θ (Degree)	2 θ (Radians)	Cos (θ)	FWHM (Degree)	FWHM (Radians)	ZnO-NPs’ Diameter (nm)
1	31.79	0.55	0.96	0.32	0.0055	26.16
2	34.45	0.60	0.96	0.31	0.0054	26.87
3	36.28	0.63	0.95	0.33	0.0057	25.49
4	47.59	0.83	0.92	0.35	0.0062	24.53
5	56.65	0.99	0.88	0.38	0.0066	23.76
6	62.93	1.10	0.85	0.42	0.0072	22.44
7	66.45	1.16	0.84	0.36	0.0063	26.40
8	68.03	1.19	0.83	0.43	0.0076	22.04
9	69.17	1.21	0.82	0.42	0.0074	22.80

## Data Availability

The original contributions presented in this study are included in the article; further inquiries can be directed to the corresponding authors.

## Supplementary Materials

The following supporting information can be downloaded at: https://www.mdpi.com/article/10.3390/molecules29225327/s1, Figure S1: Synthesis of Aspirin Anhydride; Figure S2: Synthesis of Asplatin; Figure S3: UV spectra of (A) Asp, and (B) ZnO-NPs.
