# Supplementary material for: Sustainable Nanomedicine: Enhancement of Asplatin’s Cytotoxicity In Vitro and In Vivo Using Green-Synthesized Zinc Oxide Nanoparticles Formed via Microwave-Assisted and Gambogic Acid-Mediated Processes
Source: Molecules. 2024 Nov 12;29(22):5327. doi: 10.3390/molecules29225327 (PMC11596978; doi:10.3390/molecules29225327)
Supplement: Supplementary file 1 [file molecules-29-05327-s001.zip › molecules-3269483-supplementary.pdf]

Supplementary Materials

Synthesis of Asplatin

The synthesis and characterization of asplatin was conducted as described in our previously reported study [6]. In brief, acetylsalicylic acid anhydride were synthesized, such that an equimolar amount of salicylic acid (0.014 mol, 2 g) and acetylsalicylic acid (0.014 mol, 2.5 g) were allowed to react under anhydrous conditions using acetic anhydride (0.028 mol, 2.85 g). The mixture was allowed to reflux for 1 h at a temperature range of 80 °C–100 °C, then maintaining a continually decreasing pressure over the ongoing reaction for 24 h to give a maximum absolute pressure of about 25 mmHg. The obtained product was then dissolved in an organic solvent such as dichloromethane and crystallized to get a pure yield of an acetylsalicylic acid anhydride.

Furthermore, oxoplatin was prepared via reacting 374 mg of cisplatin (1.23 mmol) suspended in 15 mL distilled water with 12.5 mL of H_2O_2 (30% *w*/*v*, 0.1 mol) added dropwise. The reaction was allowed to take place for 1 h at an increasing temperature from 50 °C to 100 °C; then, it was stirred for an extra 12 h at room temperature. The resultant was lyophilized, washed with cold water and ethanol and dried in a vacuum. Finally, asplatin was prepared by reacting a solution of oxoplatin (0.9 mmol, 300 mg) in 15 mL dimethylsulfoxide (DMSO) with the previously prepared acetylsalicylic acid anhydride (1.8 mmol, 620 mg), and the mixture was stirred for 24 h at room temperature. The obtained residue was washed with acetone and ether and dried under a vacuum. The final product of asplatin was collected as dark yellow crystals of yield 78% and characterized by different techniques.

The structural elucidation of asplatin was done by ^{1}H-NMR (400 MHz, DMSO-d6) and showed the following signals: δ 7.77–7.76 (d, 1H, ArH), 7.47–7.44 (t, 1H, ArH), 6.90–6.89 (t, 1H, ArH), 6.87–6.86 (d, 1H, ArH), 2.98 (s, 3H, NH_3), 2.52 (s, 3H, CH_3), 2.49 (s, 3H, NH_3) and 2.33 (s, 1H, OH).

The following schemes were adopted; where ***Figure S1*** was carried out for the preparation of aspirin anhydride via reacting equimolar amounts of aspirin with salicylic acid using molar excess of acetic anhydride under reduced pressure. ***Figure S2*** was then performed, where cisplatin was first converted into oxoplatin to be ready for reaction with the previously prepared aspirin anhydride to render the desired final product asplatin.

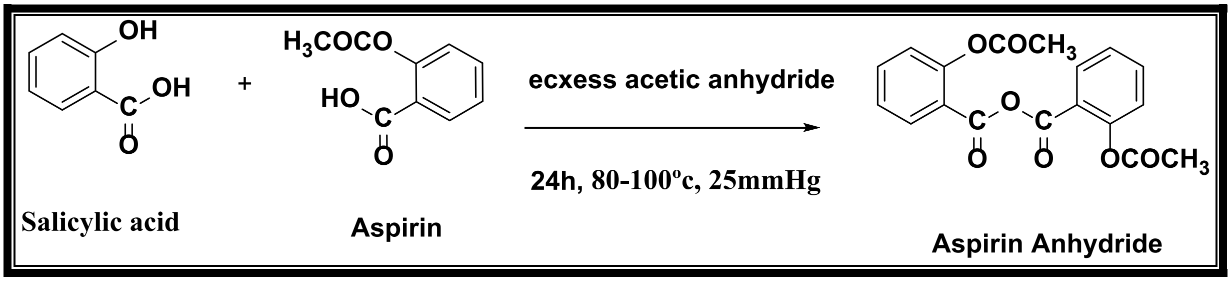

Figure S1. Synthesis of Aspirin Anhydride.

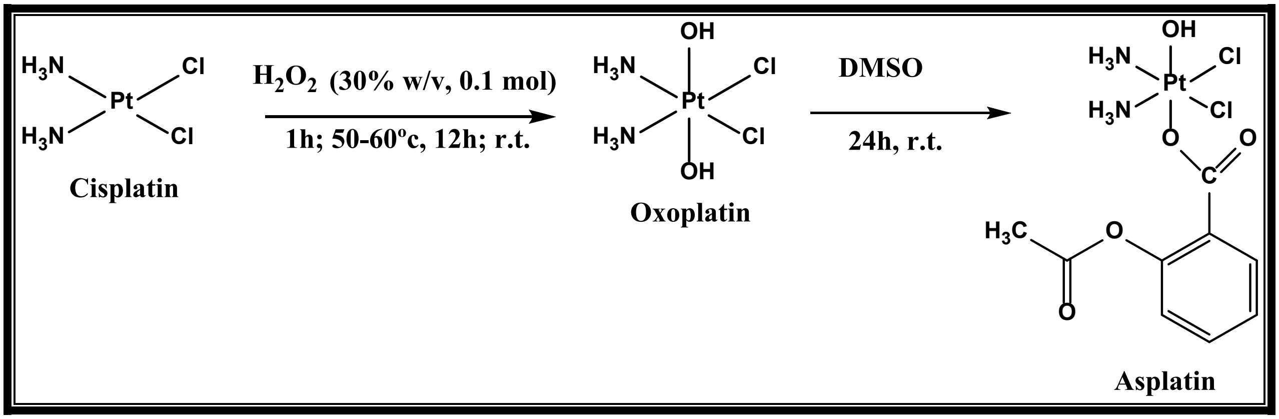

Figure S2. Synthesis of Asplatin.

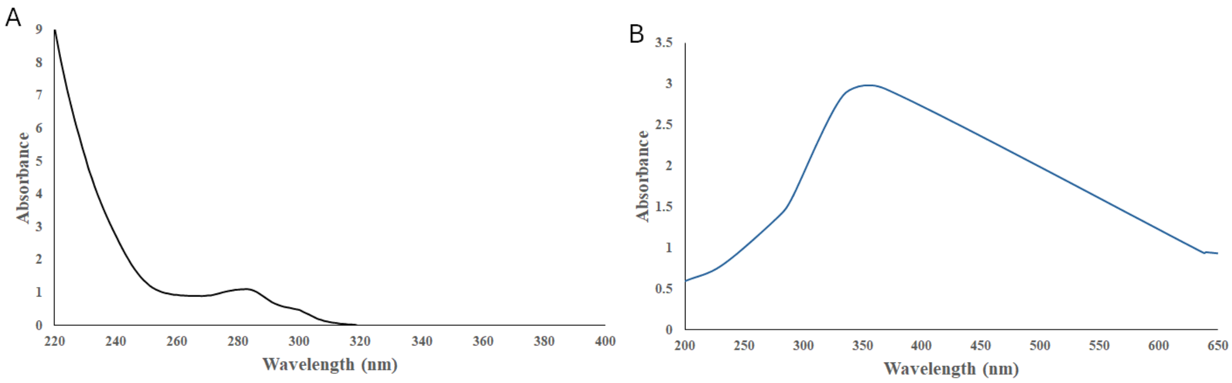

Figure S3. UV spectra of (A) Asp, and (B) ZnO-NPs.
